# Evaluation of viral respiratory pathogens in children aged under five hospitalized with lower respiratory tract infections

**DOI:** 10.14744/nci.2021.69923

**Published:** 2022-04-14

**Authors:** Gulsen Akkoc, Ceren Dogan, Suleyman Bayraktar, Kamil Sahin, Murat Elevli

**Affiliations:** 1 Division of Pediatric Infectious Diseases, Department of Pediatrics, Haseki Training and Research Hospital, Istanbul, Turkey; 2 Department of Pediatrics, Haseki Training and Research Hospital, Istanbul, Turkey

**Keywords:** Children, lower respiratory tract infections, pneumonia, real-time multiplex polymerase chain reaction, respiratory viruses

## Abstract

**Objective:**

Lower respiratory tract infections (LRTIs) are responsible for significant morbidity and mortality in children. Viral pathogens are responsible for 50–70% of LRTIs. The real-time multiplex polymerase chain reaction (RT-MPCR) tests allow the simultaneous detection of several different viruses along with some bacterial pathogens and give faster and more reliable results than viral culture. We aimed to describe the disease etiology and the clinical, laboratory, and radiological characteristics of children aged under 5 years who were hospitalized in a tertiary care medical center with LRTIs assayed using an RT-MPCR respiratory pathogen panel, and evaluate the effects of the detection of etiology on treatment and outcome.

**Methods:**

This retrospective study was conducted in the tertiary medical health center. The study group comprised all pediatric cases aged under five who were hospitalized due to LRTIs in the pediatric wards and pediatric intensive care unit (ICU) and undergone RT-MPCR analyses between January 2019 and February 2020. RT-MPCR analyses of samples from nasopharyngeal swabs were consecutively evaluated.

**Results:**

A total of 65 samples were collected from aged under 5 years who were hospitalized with LRTIs and screened for respiratory viruses. Specimens were collected from pediatric ICU (18.5%) and pediatric wards (81.5%). The overall positive rate was 89.2% (58/65). Forty of the patients (61.5%) were positive for a single pathogen, 15 (23.6%) for two, and three (4.6%) for three pathogens. The most common virus was respiratory syncytial virus (RSV) (32.3%), followed by human rhinovirus (HRV) (30.8%). In HRV-positive patients, eosinophil count was higher than that in Influenza A/B- and Human metapneumovirus-positive patients (respectively p=0.014, 0.005). In RSV-positive patients, hospitalization duration and neutrophil, lymphocyte, C-reactive protein level had moderate correlation (respectively; r=0.587; p=0.005, r=–0.436; p=0.038, r=0.498; p=0.022).

**Conclusion:**

Despite the limited number of participants from a single center, a wide range of causative pathogens were detected in our study. In addition, we found that viral pathogens are common etiologies of LRTIs. To describe the disease etiology in LRTIs, assays using an RT-MPCR respiratory pathogen panel, would be beneficial to the detection of etiology and treatment.

**L**ower respiratory tract infections (LRTIs) are responsible for significant morbidity and mortality in children [1, 2]. They are the most common childhood illnesses and account for approximately 50% of all hospitalizations of those aged under 5 [3]. LRTIs, particularly pneumonia, are the most common cause of mortality in this age group and accounted for 15% (>800,000) of deaths globally in 2017 [2, 4]. Viral pathogens are responsible for 50–70% of LRTIs [5]. Respiratory syncytial virus (RSV) is the major cause of bronchiolitis in young children, and rhinoviruses, parainfluenza viruses (PIVs), metapneumoviruses, and influenza (INF) viruses are important causes of LTRIs throughout childhood; coronaviruses and adenoviruses are less-common causes and human bocaviruses have also recently been identified as significant contributors [5–7]. Inadequate etiological diagnosis of LRTIs can lead to the unnecessary and inappropriate use of antibiotics, thereby lengthening hospital stays, increasing hospital costs, and contributing to antibiotic resistance [8]. Rapidly identifying the pathogens which involve the viral infection is crucial for early diagnosis and clinical decision-making by pediatricians [9].

Conventional diagnostic methods for LRTIs, such as viral culture, hemagglutination-inhibition assays, enzyme immunoassays, and direct fluorescent antibodies, were once the mainstay of pathogen detection; however, they are unable to determine etiological factors in patients with LRTIs. In addition, these methods are time consuming, labor-intensive, and/or operator dependent [10, 11].

The development of molecular diagnostics has made it easier to detect the etiology of pathogens in respiratory infections. The real-time multiplex polymerase chain reaction (RT-MPCR) test is more sensitive, rapid, and reliable than standard respiratory virus (RV) culture and antigen-detection methods [12, 13]. Expanded RT-MPCR panels allow the simultaneous detection of several different viruses along with some bacterial pathogens, and give faster and more reliable results than viral culture [14, 15].

The high sensitivity and expanded capability of these tests are expected to improve understanding of the epidemiology of respiratory tract infections. The widespread use of RT-MPCR will improve awareness and understanding of the viral causes of pneumonia and bronchiolitis, besides effective prevention strategies, and pathogen epidemiology in pediatric patients with LRTIs.

The current study describes the disease etiology and the clinical, laboratory, and radiological characteristics of children aged under 5 years who were hospitalized in a tertiary care medical center with LRTIs assayed using an RT-MPCR respiratory pathogen panel, and evaluates the effects of the detection of etiology on treatment and outcome.

**Highlight key points**

• The most common virus was RSV (21/65; 32.3%), followed by HRV (20/65; 30.8%).
• RSV-positive patients were significantly younger than the INFA/B-positive patients.
• In HRV-positive patients, the median eosinophil count was higher than that in INFA/B- and HMPV-positive patients.
• RSV-positive patients showed a significant difference in lymphocyte, eosinophil, and CRP levels, HRV-positive patients in lymphocyte levels, INFA/B-positive patients in neutrophil, eosinophil, and CRP levels, HPMV-positive patients in eosinophil levels between admission and after clinical improvement.
• Hospitalization duration and neutrophil count had a positive moderate correlation, in RSV-positive and INFA/B-positive patients.

## Materials and Methods

The study was carried out in accordance with the Helsinki Declaration. The Ethics Committe of Istanbul Haseki Trainning and Research Hospital approved the study protocol (Date: 21.4.2021, Number: 09-2021).

### Patients

This retrospective study was conducted in the Tertiary medical health center. The study group comprised all pediatric cases aged between 1 month and 5 years old who were hospitalized in the pediatric wards and pediatric intensive care unit (ICU) due to LRTIs and undergone RT-MPCR analyses between January 2019 and February 2020. RT-MPCR analyses of samples from nasopharyngeal swabs were consecutively evaluated.

The inclusion criteria were patients between 1 month and 5 years old with symptoms and signs of LRTIs. The exclusion criteria were antiviral treatment before admission, having immune deficiency or chronic pulmonary disease, and being older than 5 years old age. LRTIs were diagnosed clinically and defined as having at least two of the complaints following fever, cough or wheezing and crackles, and/or rhonchi on physical examination.

### Sampling and Detection of Viral Etiologies

Nasopharyngeal respiratory samples were taken via Dacron-tipped swabs within 72 h of admission and immediately placed into a sterile vial containing viral transport media. The nucleic acids were extracted using the manual silica-based membrane columns with commercial kit and tested for RVs using RT-PCR multiplex testing with the Anyplex^TM^ II RV16 detection system (Seegene, Seoul, South Korea), according to the manufacturer’s instructions. In total, 16 RVs can be detected by the Anyplex^TM^ II RV16 system including INFA/B, human PIV 1, PIV 2, PIV 3, PIV 4, human RSV A/B, human metapneumovirus (HMPV), human coronavirus (CoV) 229E, CoV NL63, CoV OC43, human enterovirus, human rhinovirus (HRV), human adenovirus, and human bocavirus (HBoV) 1/2/3/4.

### Data Collection

Demographic data, clinical and laboratory findings, treatment, hospitalization duration, ICU requirement, the day of clinical improvement, and complications of the patients were recorded retrospectively from their medical records. The laboratory results of the patients were acquired on the day of admission. Laboratory data such as complete blood count, C-reactive protein (CRP), and blood culture results were collected retrospectively at admission and after clinical improvement from the medical records.

### Statistical Analysis

Statistical analysis was performed using Statistical Package for the Social Sciences 22.0 software (IBM Corp, Armonk, NY, USA). The variables were investigated using histograms, probability plots, and analytical methods (the Kolmogorov-Smirnov/Shapiro-Wilk’s test) to determine whether they were normally distributed. Numbers and percentages were used to express categorical variables; the mean±standard deviation or the median with interquartile range (IQR) was used for numerical variables. Continuous variables were expressed according to the parametric or nonparametric distribution. Categorical variables were compared using the Chi-square test. The Mann-Whitney U test and the student test were used to compare mean or median values between two groups, depending on the sample distribution. A p<0.05 was regarded as the alpha (α) significance level. The Wilcoxon test was used to compare the changes in leukocyte, neutrophil, lymphocyte, eosinophil, and CRP values between admission and after clinical improvement. A p<0.05 was considered statistically significant. If both parameters were normally distributed, the correlation coefficients and their significance were calculated using the Pearson test, otherwise, the Spearman test was used. A 5% type-I error level was used to infer statistical significance. Pathogen-specific comparisons of demographic and laboratory findings were carried out between each mono-pathogen subgroup (defined as being solely RSV, HRV, INFA/B, or HMPV positive and having more than five patients). Comparisons of pathogens were made according to seasonal distribution (winter, spring, summer, and autumn) and when overall significance was observed, post-hoc tests were performed using the Bonferroni correction.

## Results

### Respiratory RT-MPCR Results

**Figure 1.**
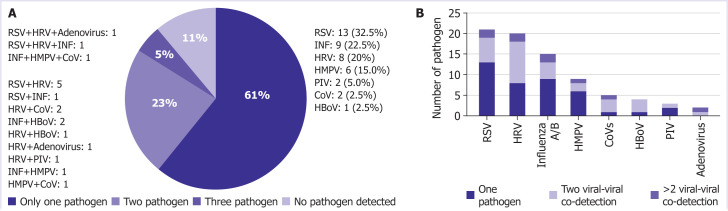
The detailed distribution of the viral etiologies **(A)** according to pathogen co-detection number, **(B)** according to specific pathogens. CoVs: Human coronavirus 229E/NL63/OC43; HBoV: Human bocavirus; HMPV: Human metapneumovirus; HRV: Human rhinovirus; INF: Influenza A/B; PIV: Parainfluenza virus; RSV: Respiratory syncytial virus.

According to the inclusion criteria, between January 2019 and February 2020 a total of 65 samples were collected from 65 patients aged between 1 month and 5 years who were hospitalized with LRTIs and screened for RVs. Specimens were collected from the pediatric ICU (n=12; 18.5%) and pediatric wards (n=53; 81.5%). The overall positive rate was 89.2% (58/65). Only seven (10.8%) patients were negative for detectable viral pathogens. Forty of the patients (61.5%) were positive for a single pathogen, 15 (23.6%) were positive for two pathogens, and three (4.6%) were positive for three pathogens. The most common virus was RSV (21/65; 32.3%), followed by HRV (20/65; 30.8%). Among the 65 samples, 53 (81.5%) were taken in the winter and autumn seasons. All RSV-positive patients were seen in the winter season (p=0.003). The incidence of HMPV-positive patients was significantly higher in the autumn (62.6%) than in the other seasons (the values for winter, spring, and summer were 8.9%, 0%, and 0%, respectively; p<0.000). The incidence of CoVs was significantly higher in spring (40.0%) than in winter (6.6%; p=0.018). The detailed distribution of the viral etiologies is shown in Figures 1a and 1b.

### Demographic and Clinical Features

**Figure 2.**
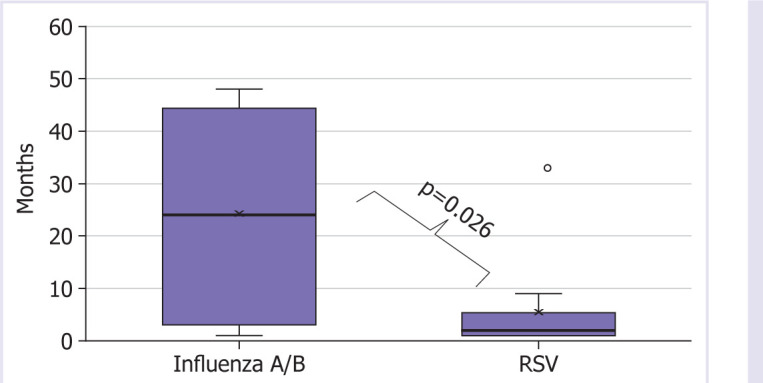
Comparison of age between RSV and Influenza A/B-positive patients. RSV: Respiratory syncytial virus.

The mean age of the patients was 12.43±14.83 months (1–58 months), the median age was 6 months (IQR: 13.5 months), and 48 of the patients (73.8%) were male. Overall, 52 (80%) of the cases were under 2 years of age. Although the median age of the girls was lower than that of the boys, there was no statistically significant difference between the two groups (3 months [IQR: 17 months] vs. 6 months [IQR: 12 months]; p=0.393). There was no significant difference in the etiological distribution of viral pathogens according to gender. The RSV-positive patients were significantly younger than the RSV-negative patients (2 months [IQR: 5 months] vs. 8.5 months [IQR: 20 months]; p<0.000). The HRV-positive patients were significantly younger than the HRV-negative patients (4 months [IQR: 5 months] vs. 8 months [IQR: 21 months]; p=0.011). The INFA/B- and HBoV-positive patients were significantly older than the INFA/B- and HBoV-negative patients (15 months [IQR: 47 months] vs. 5.5 months [IQR: 10 months]; p=0.034; and 48 months [IQR: 31 months] vs. 6 months [IQR: 47 months]; p=0.003, respectively). The RSV-positive patients were significantly younger than the INFA/B-positive patients (2 months [IQR: 5 months] vs. 24 months [IQR: 42 months]; p=0.026) (Fig. 2). The comparison of the viral etiologies according to age is summarized in Table 1.

**Table 1.** The comparison of the viral etiologies according to age

	Age, months Median (IQR)		p
	Positive	Negative	
RSV (n=21)	2 (5)	8.5 (20)	<**0.000**
HRV(n=20)	4 (5)	8 (21)	**0.011**
Influenza A/B (n=15)	15 (33)	5.5 (10)	**0.034**
HMPV (n=9)	8 (11)	6 (19)	0.613
CoVs (n=5)	7 (9)	6 (18)	0.739
HBoV (n=4)	48 (31)	6 (12)	**0.003**
PIV (n=3)	14^†^	6 (13)	0.252
Adenovirus (n=2)	2 (0)	6 (14)	0.170
No pathogen detected (n=7)	13 (15)	6 (13)	0.081
Total (n=65)	6 (13.5)		

*: Significance value as <0.05; used Mann-Whitney U test; IQR: Interquartile range; †: IQR was not customized; CoVs: Human coronavirus 229E/NL63/OC43; HBoV: Human bocavirus; HMPV: Human metapneumovirus; HRV: Human rhinovirus; PIV: Parainfluenza virus; RSV: Respiratory syncytial virus.

Among the participants, 20% (n=14) had underlying chronic diseases. The most common of these were prematurity (n=3; 4.3%), motor-mental retardation (n=3; 4.3%), and congenital heart diseases (n=3; 4.3%). Cough was the most common symptom among the patients (n=60; 92.3%). Wheezing was the second most common symptom (n=57, 87.7%). Fever was seen only in 32.3% (n=21) of the patients. According to the viral etiologies, fever was significantly common in INFA/B-positive patients (66.7% vs. 22.0%; p=0.003). The frequency of symptoms according to the viral etiologies is summarized in Table 2.

**Table 2.** The frequency of symptoms according to the viral etiologies

	Cough		p*	Wheezing		p*	Fever		p*
	Yes (%)	No (%)		Yes (%)	No (%)		Yes (%)	No (%)
RSV (n=21)	85.7	14.3	0.318	95.2	4.8	0.259	19.0	81	0.114
HRV (n=20)	100	0	0.313	95.0	5.0	0.417	10.0	90.0	**0.011**
Influenza A/B (n=15)	93.3	6.7	1	80.0	20.0	0.373	66.7	33.3	**0.003**
HMPV (n=9)	88.9	11.1	0.538	66.7	33.3	0.073	44.4	55.6	0.455
CoVs (n=5)	100	0	1	80.0	20.0	0.493	20.0	80.0	1
HBoV (n=4)	100	0	1	100	0	1	50.0	50.0	0.589
PIV (n=3)	100	0	1	66.7	33.3	0.330	33.3	66.7	1
Adenovirus (n=2)	100	0	1	100	0	1	0	100	1
No pathogen detected (n=7)	100	0	1	80.0	20.0	0.493	60.0	40.0	0.318

CoVs: Human coronavirus 229E/NL63/OC43; HBoV: Human bocavirus; HMPV: Human metapneumovirus; HRV: Human rhinovirus; PIV: Parainfluenza virus; RSV: Respiratory syncytial virus.

### Laboratory Findings at Admission and Comparison of Admission and Follow-up Laboratory Findings

**Figure 3.**
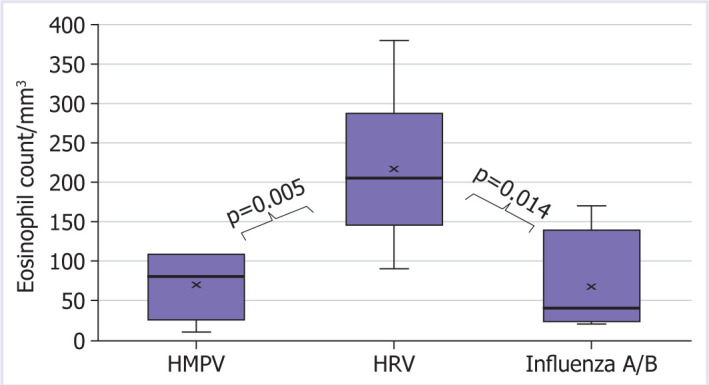
Comparison of median eosinophil count between, HRV, Influenza A/B, and HMPV-positive patients. HMPV: Human metapneumovirus; RSV: Respiratory syncytial virus.

Blood culture was taken in all patients and none yielded pathogens. Among the participants, the mean leukocyte count was 11.002±3.751/mm^3^ (2.600–21.790/mm^3^), the median neutrophil count was 4.440/mm^3^ (IQR: 3938/mm^3^), the median lymphocyte count was 4.440/mm^3^ (IQR: 53.183/mm^3^), the median eosinophil count was 90/mm^3^ (IQR: 170/mm^3^), and the median CRP level was 8.90 mg/dl (IQR: 25.90 mg/dl). The eosinophil count was significantly higher in HRV-positive patients than in HRV-negative patients (p=0.016). The CRP level was significantly higher in INFA/B-positive patients than in INFA/B-negative patients (p=0.012). The neutrophil count was significantly higher and the lymphocyte count was significantly lower in HBoV-positive patients than in HBoV-negative patients (p=0.020 and 0.026, respectively). The detailed laboratory findings according to viral etiology are summarized in Table 3. Comparing the mono-pathogens showed that the median leukocyte count in RSV-positive patients was lower than that is HRV-positive patients (p=0.082), although this was not statistically significant. In HRV-positive patients, the median eosinophil count was higher than that in INFA/B- and HMPV-positive patients (p=0.014 and 0.005, respectively) (Fig. 3).

**Table 3.** The detailed laboratory findings according to viral etiologies

	Leukocyte/mm^3^		Neutrophil/mm^3^		Lymphocyte/mm^3^		Eosinophil/mm^3^		C-reactive protein mg/dl
	Mean±SD (Min–Max)	p*	Median (IQR)	p*	Median (IQR)	p*	Median (IQR)	p*	Median (IQR)	p*
RSV (n=21)										
	10176±2390		3520		4870		120		8.60	
	(6210–15360)	0.143	(3470)	0.064	(3025)	0.293	(210)	0.148	(23.30)	0.400
	11487±4224		5050		4440		70		10.20	
	(2600–21790)		(4690)		(3070)		(155)		(29.4)	
HRV (n=20)										
	11455±3649		3335		4515		165		11.10	
	(6210–18620)	0.521	(4540)	0.572	(3083)	0.653	(148)	**0.016**	(33.8)	0.126
	10866±3873		4880		4320		50		7.80	
	(2600–21760)		(3080)		(2860)		(160)		(16.20)	
Influenza A/B (n=15)										
	11094±4947		5955		3575		60		28.65	
	(2600–21760)	0.901	(4340)	0.531	(4203)	0.244	(165)	0.412	(16.40)	**0.012**
	11041±3445		4520		4460		90		8.40	
	(5730–18620)		(3905)		(2930)		(185)		(16.40)	
HMPV (n=9)										
	11502±3717		4470		5460		70		14.30	
	(5800–17740)	0.670	(2025)	0.622	(5585)	0.220	(90)	0.510	(25.10)	0.608
	10978±3825		4745		4330		90		8.75	
	(2600–21760)		(4115)		(2755)		(188)		(29.50)	
CoVs (n=5)										
	11274±2923		4470		5460		130		10.20	
	(7960–14760)	0.868	(4265)	0.736	(3025)	0.255	(175)	0.799	(41.60)	0.622
	11033±3869		4625		4330		85		9.15	
	(2600–21790)		(3900)		(3243)		(173)		(25.70)	
HBoV (n=4)										
	12610±3326				2250		40		20.60	
	(9820–17340)	0.380		**0.020**	(2433)	**0.026**	(75)	0.120	(11.00)	0.148
	10947±3815		4470		4460		90		8.60	
	(2600–21790)		(3750)		(3050)		(170)		(31.20)	
PIV (n=3)										
	10533±4540		6340		4440		50		1.60	
	(7480–15750)	0. 827	(^†^)	0.440	(^†^)	0.354	(^†^)	0.651	(^†^)	0.879
	11079±3786		4540		4390		90		9.80	
	(2600–21790)		(3733)		(2948)		(175)		(25.90)	
Adenovirus (n=2)										
	10480±2192		3315		5870		100		23.00	0.879	
	(8930–12030)	0.843	(^†^)	0.440	(^†^)	0.354	(^†^)	0.651	(^†^)	
	11071±384		4690		4340		90		9.40	
	(2600–21760)		(3980)		(3015)		(165)		(25.00)	
No pathogen detected (n=7)										
	10553±4550		5165		4080		40		25.30	
	(5730–15350)	0.515	(7483)	0.779	(2078)	0.620	(218)	0.504	(106.1)	0.464
	11086±3771		4560		4440		90		8.90	
	(2600–21790)		(3450)		(3220)		(170)		(24.50)	

*: Significance value as: <0.05 (the student or the Mann-Whitney U tests were used to compare mean or median values between two groups, depending on the sample distribution). IQR: Interquartile range; †: IQR was not customized; CoVs: Human coronavirus 229E/NL63/OC43; HBoV: Human bocavirus; HMPV: Human metapneumovirus; HRV: Human rhinovirus; PIV: Parainfluenza virus; RSV: Respiratory syncytial virus.

**Figure 4.**
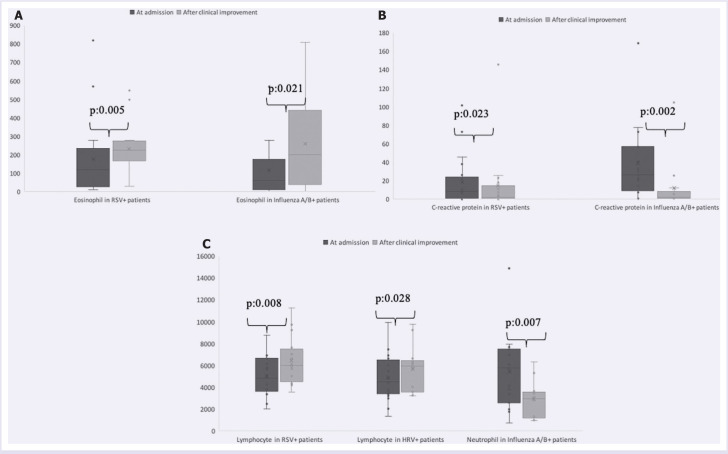
Significant differences at the laboratory level between admission and after clinical improvement, **(A)** eosinophil level difference in RSV-positive and Influenza A/B-positive patients, **(B)** C-reactive protein level difference in RSV-positive and Influenza A/B-positive patients, **(C)** lymphocyte level difference in RSV-positive and HRV-positive patients, and neutrophil level difference in Influenza A/B-positive patients. HRV: Human rhinovirus; RSV: Respiratory syncytial virus.

Fifty patients had undergone a complete blood count and CRP analysis at admission and after clinical improvement. RSV-positive patients showed a significant difference in lymphocyte, eosinophil, and CRP levels between admission and after clinical improvement (p=0.008, 0.005, and 0.023, respectively). HRV-positive patients showed a significant difference in lymphocyte levels between admission and after clinical improvement (p=0.028). INFA/B-positive patients showed a significant difference in neutrophil, eosinophil, and CRP levels between admission and after clinical improvement (p=0.007, 0.021, and 0.002, respectively). HPMV-positive patients showed a significant difference in eosinophil levels between admission and after clinical improvement (p=0.021). All significant differences at the laboratory level are shown in Figure 4a–c.

### Management

All patients had received antibiotic therapy, and oseltamivir treatment was given to INFA/B-positive patients. Clinical improvement was seen in all patients and all were discharged from hospital with a good clinical outcome. The mean hospitalization duration was 9.45±6.35 days (3–42 days) and the median hospitalization duration was 8 days (IQR: 3.50 days). Clinical improvement day data were available for 63.1% (n=41) of the patients. The median clinical improvement day was the 5^th^ day (IQR: 4^th^ day) and the mean clinical improvement day was 6.02±3.45 (2–20). Among the 18.5% (n=12) of patients who had an ICU requirement, four patients (33.3%) underwent mechanic ventilation and three patients (25.0%) underwent high-flow noninvasive ventilation. The viral etiologies seen in ICU patients as follows: RSV, INF, HRV, RSV-HRV co-detection, INF-HBoV co-detection, and RSV-HRV-INF co-detection, respectively, in four patients, two patients, two patients, two patients, one patient, and one patient.

In RSV-positive patients, age and CRP had a positive moderate correlation (r=0.572; p=0.007). Hospitalization duration and neutrophil count had a positive moderate correlation (r=0.587; p=0.005), lymphocyte count had a moderate negative correlation (r=–0.436; p=0.038) and CRP level had a positive moderate correlation (r=0.498; p=0.022). Clinical improvement day and neutrophil count had a positive moderate correlation (r=0.596; p=0.015), leukocyte count had a positive moderate correlation (r=0.538; p=0.032) and CRP level had a positive moderate correlation (r=0.510; p=0.044). In INFA/B-positive patients, age and lymphocyte count had a strong negative correlation (r=–0.731; p=0.002), and hospitalization duration and neutrophil count had a moderate positive correlation (r=0.516; p=0.049). In HMPV-positive patients, age and lymphocyte count had a strong negative correlation (r=–0.733; p=0.025) and leucocyte count had a good correlation (r=0.683; p=0.042).

## Discussion

This study used the Anyplex^TM^ II RV16 detection respiratory viral panel assay to detect 16 RVs in 65 samples from children hospitalized with LRTIs who were younger than 5 years old. In our study, at least one pathogen was detected in 89.2% of the samples; this high positivity rate provided important information about LTBIs in patients aged under 5 years, despite the limited number of participants. Viral pathogens are more commonly associated with LTBIs in children younger than 5 years of age [5, 16–18]. Bacterial infections made up 15% of the LTBIs [5, 16–19]. Notably, children younger than 2 years old showed a higher rate of viral infections. The higher rate of viral pathogens detected in our study compared with other reports may be explained by the fact that all the patients in our study were younger than 5 years old and the majority were younger than 2 years. In addition, the RT-PCR method used in our study could not detect common bacterial pathogens in the respiratory tract such as Mycoplasma pneumoniae and Bordetella pertussis [16–18]. Viral infections are often difficult to distinguish from bacterial infections based solely on clinical, laboratory, or radiological findings and specific diagnosis is largely based on microbiological findings, particularly at the molecular level [20].

In terms of the distribution of viral etiologies, RSV was the most common pathogen in our study, with notable seasonal distributions in the winter and autumn months. RSV is generally considered to be the most frequent pathogen in pediatric LRTIs, particularly bronchiolitis, according to the published literature, and our results were consistent with this [5, 21]. Also, RSV is a more significant etiologic cause of LRTIs in infancy compared with in other age groups [5]. HRV was the second most common pathogen in our study. The Etiology of Pneumonia in the Community study of a large population revealed that HRV was seen nearly as frequently as RSV, similar to in our study [5]. The widespread use of molecular diagnostic techniques has provided additional information about the role of HRV in LRTIs, and it is considered to be one of the common causes of pneumonia [5]; however, HRV has also been detected in asymptomatic controls at a rate similar to its detection in cases of pneumonia.

In our study, the viral-viral coinfection rate was 28.0% and mostly involved coexisting HRV and RSV. In the literature, the reported rate of viral–viral coinfection in LRTIs ranges between 19.8% and 42.5% as measured with various RT-MPCR assays [19, 22]. In our study, the finding that HRV was the most common virus in viral–viral coinfections may have been because of its asymptomatic existence. HBoV was generally present along with another viral pathogen rather than being the sole pathogen in our study. Knowledge of the epidemiology of HBoV in LRTIs is inadequate due to limited studies. However, it has been reported that HBoV is commonly detected in co-infections and often shows asymptomatic colonization [23]. This makes it difficult to accurately assess HBoV-related LRTIs.

In our study, 73.8% of the patients were male and there was no significant difference in the distribution of RVs according to gender, which was consistent with other reports [19, 22, 24, 25]. Our analysis of the distribution of RVs according to age revealed that RSV and HRV were seen in younger patients and INFA/B and HBoV were seen in older patients. Moreover, the RSV patients were significantly younger than the INFA/B patients. A previous study with a large number of participants conducted by Jain et al. [5] revealed that RSV and HRV were more common in children younger than 2 years old and their incidence decreased sharply with increasing age. In addition, INFA/B was far more common in older participants [5].

Currently available inflammatory biomarkers, such as leukocyte count, CRP, and procalcitonin, reflect the host inflammatory response to infection. However, these biomarkers are insufficient diagnostic assays for predicting etiology in LRTIs [26]. Notably, in our study, the eosinophil count was significantly higher in HRV-positive patients than in HRV-negative patients. Eosinophils have potential antiviral activity via the expression of different molecules during viral recognition [27]. Also, it has been reported that eosinophils bind to HRV16 using the intercellular adhesion molecule-1 *in vitro* [28]. Thus, the higher eosinophil level in HRV may be related to be its antiviral mechanism. In addition, in RSV-positive and INFA/B-positive patients, there were significant differences in eosinophil levels at the time of admission and after clinical improvement. Lindemans et al. [29] revealed the systemic eosinophil response induced by RSV and showed that the eosinophil numbers had increased significantly in patients at 6 weeks. This also demonstrated the antiviral activity of eosinophils in viral infections. In our study, none of the patients died during the hospitalization. Although pneumonia is a leading cause of mortality in children under 5 years old, mortality-attributed LRTIs are uncommon in developed countries or in places with easily accessible medical or healthcare facilities [1, 30].

Community-acquired pneumonia is the second most costly and fifth most common reason for hospitalization in children [31]. As mentioned above, viral infections are difficult to distinguish from bacterial infections based solely on clinical, laboratory, or radiological findings, which may lead to unnecessary antimicrobial use [32]. This causes additional concern about the emergence and spread of multidrug-resistant bacteria. However, viral pathogens are more common in LRTIs, and empirical antibiotic therapy has to be initiated frequently in patients due to the late results from conventional culture and serological methods [33, 34]. The early and accurate detection of etiological agents and the initiation of appropriate treatment significantly reduce mortality and morbidity [35]. Molecular tests that can quickly identify various viruses simultaneously can help to initiate appropriate therapy [36]. RT-MPCR tests are rapid and more sensitive and specific tests than conventional methods for use with respiratory secretions; given these abilities, they could support the management of hospitalized patients and improve surveillance for viral infections [37, 38]. RT-MPCR methods have demonstrated qualitative detection and high diagnostic accuracy with more than 90.0% sensitivity, and different panel assays have usually been comparable in the detection of viral pathogens [39, 40]. In addition to the recognized features of these tests in diagnosis, more research is needed on their effects in terms of preventing unnecessary antibiotic usage and hospital costs.

As mentioned above, empirical antibiotic therapy was initiated frequently in LRTIs. Ferronato et al. [41] found that the identification of RSV (using immunofluorescence assays in nasopharyngeal aspirates) in infants was associated with the discontinuation of antibiotics. Unfortunately, we could not obtain scientifically data about the effect of the clarification of the viral etiology on antimicrobial treatment discontinuation in our study. Therefore, we could not demonstrate this subject in our study. This was one of the major limitations of our study.

There were several limitations to our study. First, all data were obtained from a single center and so may not be representative of the entire pediatric population. Second, although there was a high rate of detected viruses, the sample size was relatively small, so further studies with large numbers of participants are needed to confirm these observations. Third, our RT-MPCR assay was not able to detect common bacterial respiratory pathogens.

### Conclusion

In conclusion, despite the limited number of participants from a single center, a wide range of causative pathogens were detected in our study. In addition, we found that viral pathogens are common etiologies of LRTIs, especially in the winter and autumn seasons, and RSV was the most common among these viral pathogens. To describe the disease etiology in LRTIs, assays using an RT-MPCR respiratory pathogen panel, would be beneficial to the detection of etiology and treatment.
